# Enhancing the efficacy of albendazole for liver cancer treatment using mesoporous silica nanoparticles: an *in vitro* study

**DOI:** 10.17179/excli2021-4491

**Published:** 2022-01-11

**Authors:** Mohsen Ghaferi, Warda Zahra, Azim Akbarzadeh, Hasan Ebrahimi Shahmabadi, Seyed Ebrahim Alavi

**Affiliations:** 1 Department of Microbiology, School of Medicine, Rafsanjan University of Medical Sciences, Rafsanjan, Iran; 2 Nishtar Medical University and Hospital, Multan 60000, Pakistan; 3 Department of Pilot Nanobiotechnology, Pasteur Institute of Iran, Tehran, Iran

**Keywords:** Albendazole, cancer, drug delivery, MCM-41, mesoporous silica nanoparticles

## Abstract

The present study aimed to synthesize albendazole (ABZ)-loaded Mobil Composition of Matter No. 41 (MCM-41 NPs) to increase the efficacy of the drug against liver cancer. ABZ was loaded into MCM-41 NPs, and after *in vitro* characterization, such as size, size distribution, zeta potential, morphology, chemical composition, thermal profile, drug release, surface and pore volume, and pore size, their biological effects were evaluated using 3-[4,5-dimethylthiazol-2-yl]-2,5 diphenyl tetrazolium bromide (MTT) cell migration assays. The results demonstrated that monodispersed and spherical NPs with a size of 220 ± 11.5 and 293 ± 8.7 nm, for MCM-41 NPs and ABZ-loaded MCM-41 NPs, respectively, and drug loading efficiency of 30 % were synthesized. ABZ was loaded physically into MCM-41 NPs, leading to a decrease in surface volume, pore size, and pore volume. Also, MCM-41 NPs could increase the cytotoxicity effects of ABZ by 2.9-fold (IC_50_ = 23 and 7.9 µM for ABZ and ABZ-loaded MCM-41 NPs, respectively). In addition, both ABZ and ABZ-loaded MCM-41 NPs could restrain the cell migration by 12 %. Overall, the results of the present study suggest evaluating the potency of MCM-41 NPs, as a potent nanoplatform, for ABZ delivery *in vivo* environment.

See also the Graphical Abstract(Fig. 1).

## Introduction

Liver cancer is the fifth most common cancer and second cancer in terms of mortality in the world (Li et al., 2019[42]; Momenimovahed and Salehiniya, 2019[49]). The number of new cases and deaths from liver cancer was 841,080 and 781,631, respectively, in 2018 (Bray et al., 2018[12]). Liver cancer has a poor prognosis, mainly due to the diagnosis in many cases is made late or inaccurate (IARC, 2022[31]; Jiao et al., 2019[34]; Marzbani et al., 2019[47]). The five-year survival rate in liver cancer is poor and is less than 20 % (Fang et al., 2017[21]). Moreover, the recurrence rate of liver cancer following surgery is high equal to 70-80 % (De Cicco et al., 2019[15]; Du et al., 2014[18]). Various types of treatment have been used for liver cancer, including surgical resection and chemotherapy (Rabinel et al., 2017[58]).

Albendazole (ABZ) is a benzimidazole carbamate with anthelmintic activity. It functions through targeting and destruction of the β-tubulin structure to restrain the microtubule polymerization (Liu et al., 2020[43]). Recent studies have demonstrated that ABZ can restrain microtubule polymerization, and this can cause anti-tumor activity against various tumors, such as liver cancer (Liu et al., 2020[43]; Pourgholami et al., 2001[57]). Moreover, scientists have demonstrated that ABZ can cause a significant decrease in the activity of hypoxia-inducible factor-Iα (HIF-1α) and vascular endothelial growth factor (VEGF) in ovarian cancer that restrains tumor angiogenesis, and as a result, decreasing the symptoms of ascites (Noorani et al., 2015[52]). ABZ has also been found as a radiosensitizer in tumor models (e.g., metastatic melanoma and small cell lung cancer) as it can induce DNA damage (Patel et al., 2011[54]).

Despite the wide range of applications, the use of ABZ is restricted owing to low water solubility (2.7 µM), which leads to negligible or variable bioavailability following administration (Deodhar et al., 2017[16]). Many efforts have been made to overcome this barrier, such as the use of various solvents (Ibrahim et al., 2020[32]), solid dispersion (Maleki et al., 2017[46]), and microparticles and nanoparticles (NPs). Among all of these strategies, the most successful ones are developing the formulations of ABZ using nanocomplexes and NPs (Deodhar et al., 2017[16]).

NPs are potent to enhance the efficacy of drugs (Alavi and Ebrahimi Shahmabadi, 2021[6]; Alavi et al., 2013[8], 2014[9]; Movahedi et al., 2014[50]) and reduce their adverse side effects (Alavi and Ebrahimi Shahmabadi, 2021[7]; Ghaferi et al., 2020[22]; Shahabi et al., 2014[64]). Various NPs have been used for the delivery of anticancer drugs (Ebrahimi Shahmabadi et al., 2014[19]; Koohi Moftakhari Esfahani et al., 2014[37], 2018[38]). In this regard, mesoporous silica NPs (MSNPs) have drawn considerable attention as controlled drug delivery systems (Mahmood et al., 2020[45]).

**Figure 1:**
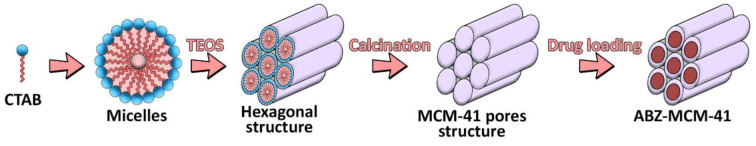
Schematic diagram of the mechanism of albendazole (ABZ)-Mobil Composition of Matter No. 41 (MCM-41) NP synthesis. N-cetyltrimethylammonium bromide (CTAB) is a cationic surfactant micelle and functions as a template for directing the polymerization of silica component (tetraethylorthosilicate; TEOS). The resulting mixture (CTAB+TEOS) is then crystallized under the hydrothermal condition, and the solid product (hexagonal structure) is generated. The solid product is then calcinated to remove CTAB, resulting in the final product (MCM-41 pores structure). The product is then loaded with ABZ, and ABZ-MCM-41 NPs are produced.

MSNPs are solid materials containing hundreds of arranged empty mesopores with a high capacity of loading for hydrophobic and hydrophilic drugs (Rahikkala et al., 2018[59]). They are cost-effective nanomaterials with low toxicity, tunable pore size, and simple fabrication method (Li et al., 2019[41]; Raza et al., 2019[62]). Their surface can be readily functionalized (Deodhar et al., 2017[16]) to improve their cellular uptake. Due to several attractive properties, such as large surface area and pore volume, as well as long regular pore structure, MSNPs are able to improve drug solubility. Also, these carriers, compared to various other polymers utilized to increase drug solubility, have demonstrated higher thermal resistance, higher levels of resistance to pH, and superior stability on storage. The porous structure of MS materials allows them to confine and stabilize the amorphous state of drug molecules within the pores rather than the crystalline state, resulting in an increase in the solubility rate of the drug molecules (Adrover et al., 2020[2]; Ghaferi et al., 2021[24]). To synthesize MSNPs, a surfactant micelle is used as a template to direct the polymerization of silica components (see graphical abstract, Figure 1(Fig. 1)) (Mehmood et al., 2017[48]). MSNPs are successfully utilized for oral delivery of hydrophobic drugs, where they can cause an improvement in the dissolution rate and bioavailability of these drugs (Koohi Moftakhari Esfahani et al., 2021[40]; Tawfeek et al., 2019[66]).

The low water solubility of drugs causes their low absorption, and as a result, their low bioavailability (Chaudhari and Handge, 2020[13]), which, in turn, can lead to a significant decrease in the therapeutic response and an increase in the overall dose (Abu-Huwaij, 2018[1]). ABZ, due to low water solubility, has low bioavailability after oral administration (Pavan Kumar et al., 2007[55]; Savio et al., 1998[63]). The present study aimed to provide a nanoplatform of ABZ using MSNPs with an enhanced therapeutic response against liver cancer. For this purpose, ABZ was loaded into the Mobil Composition of Matter No. 41 (MCM-41) NPs, which are high-ordered mesoporous materials (Yao et al., 2001[69]). Next, the *in vitro* characterization of ABZ-loaded NPs was conducted in terms of size, size distribution, zeta potential, morphology, chemical bonds, specific surface area, thermal stability using dynamic light scattering (DLS), scanning (SEM) and transmission (TEM) electron microscopy, Fourier transform-infrared (FT-IR), Brunauer-Emmett-Teller (BET), differential scanning calorimetry (DSC) and thermogravimetric analysis (TGA) methods, respectively. The efficacy of ABZ either in the encapsulated form in MCM-41 NPs or free form was evaluated on HepG2 cells using 3-[4,5-dimethylthiazol-2-yl]-2,5 diphenyl tetrazolium bromide (MTT) and cell migration assays.

## Materials and Methods

N-cetyltrimethylammonium bromide (CTAB), dimethylsulfoxide (DMSO), tetraethylorthosilicate (TEOS), acetone, sodium hydroxide (NaOH), phosphate-buffered saline (PBS), MTT, and ABZ were purchased from Merck (Darmstadt, Germany). Roswell Park Memorial Institute (RPMI)-1640 medium, Pen/Strep antibiotics, and fetal bovine serum (FBS) were purchased from Gibco (Waltham, MA, USA). Human liver cancer HepG2 cells were supplied from the Pasteur Institute of Iran (Tehran, Iran).

### Synthesis of MCM-41

To synthesize MCM-41 NPs, 1 g of CTAB was added to 480 mL MilliQ water and stirred (700 RPM, room temperature) to obtain a clear solution. Next, NaOH solution (3.5 mL, 2 M) was added (1 mL/min) to the solution, and the mixture was heated to 80 ℃ using an oil bath under continuous stirring. While heating, 6.7 mL of TEOS was added (1 mL/min) to the mixture, stirred (700 RPM, 2 h), and vacuum filtered. The solution was then washed with MilliQ water three times, and to dry the resulting precipitate, it was put in an oven at 60 ℃ overnight. The dried precipitate was crushed and heated to 550 ℃ for 5 h using a muffle furnace to remove any remaining surfactant (CTAB).

### Drug loading into MCM-41

Forty milligrams of ABZ were dissolved in 50 mL of acetone using a bath sonicator (50 Hz, 5 min). Next, 160 mg of MCM-41 NPs was added to the mixture and stirred (overnight, room temperature). The organic solvent was then removed using a rotary evaporator (40 ℃). The drug-loaded nanoparticles were scratched off from the bottom of the vessel.

### Characterization of nanoparticles

#### Dynamic light scattering

The size, size distribution, and zeta potential of MCM-41 and ABZ-loaded MCM-41 NPs were determined using Zetasizer instrument (Malvern, UK). For this purpose, a suspension of both nanoparticles (100 µg/mL) was provided in PBS (pH 7.4) using a bath sonicator (10 min), and the suspensions were introduced to the instrument.

#### Transmission electron microscopy

The morphology of ABZ-loaded MCM-41 NPs was studied using TEM (Alavi et al., 2021[4]) (Zeiss, EM10C, 80 kV, Oberkochen, Germany). For this purpose, 20 µL of the nanoparticles suspension was placed on a copper grid and imaged.

#### Scanning electron microscopy

The morphology of ABZ-loaded MCM-41 NPs was evaluated using SEM XL30 Philips (Eindhoven, The Netherlands) in a high vacuum mode. The nanoparticles were first coated with gold and then evaluated by the SEM instrument.

#### Fourier transformed infrared spectroscopy

The structure and chemical composition of MCM-41 NPs before and after loading with ABZ were characterized by Fourier-transform infrared spectroscopy (FTIR) (Bruker FRA model 106/5). The KBr tablets with KBr:ABZ, KBr:MCM-41 NPs, and KBr:ABZ-loaded MCM-41 NPs ratio of 100:1 were provided, and the related FTIR spectra were obtained in a frequency range of 400-4000 cm^-1^.

#### Thermogravimetric analysis and differential scanning calorimetry

Five milligrams of ABZ, MCM-41 NPs, and ABZ-loaded MCM-41 NPs were characterized in terms of thermal properties and thermal composition using a TGA/DSC Instruments LINSIES STP PT-1000 with a heating rate of 10 ℃/min and under an air ambient.

#### Drug release from nanoparticles

ABZ-loaded MCM-41 NPs (equal to 0.3 mg ABZ) were suspended in 5 mL PBS (pH 1.9 and 7.4) and stirred at 200 RPM at room temperature. At various time intervals (0.25, 0.5, 1, 2, 4, 6, 8, 10, 12 h), 50 µL was taken out and centrifuged. The drug concentrations in the samples were measured using the high-performance liquid chromatography (HPLC) method. Next, the cumulative drug release was calculated using the following equation:


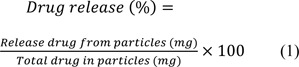


The kinetics of the drug release was then determined using mathematical models, including zero-order, first-order, Higuchi and Korsmeyer-Peppas models (Ramezani Farani et al., 2020[61], 2019[60]).

#### Brunauer-Emmett-Teller surface area analysis

The total surface area and pore size distribution of MCM-41 NPs before and after loading with ABZ were measured by a surface area and pore size analyzer (BELsorp-mini II, Japan) and Barrett-Joyner-Halenda (BJH) methods. For this purpose, 70 mg of MSNPs and ABZ-loaded MCM-41 NPs were degassed prior to analysis. BET surface area and pore size were then measured by N_2_ adsorption using a surface area and pore size analyzer (BELsorp-mini II, Japan).

### Biological effects of the nanoparticles

#### Cytotoxicity studies

The cytotoxicity effects of ABZ-loaded MCM-41 NPs compared to ABZ were evaluated using an MTT assay, as described previously (Alavi et al., 2020[10]). For this purpose, HepG2 cells were cultured in RPMI-1640 medium supplemented with 10 % FBS and 1 % penicillin/streptomycin antibiotics in a 96-well plate at a density of 10^4^ cells/well. After 24 h incubation in a humidified incubator (37 ℃, 5 % CO_2_), the media were discarded, and the cells were treated with ABZ and ABZ-loaded MCM-41 NPs at the drug concentrations of 0, 3.1, 6.3, 12.5, 25, 50, and 100 µM prepared from 250 μM stocks of ABZ in 5 % v/v DMSO-water. The plates were incubated (5 % CO_2_, 37 ℃) for 24 h, and the media were replaced with MTT solution (100 µL, 0.5 mg/mL PBS). After 4 h incubation (5 % CO_2_, 37 ℃), the MTT solution was removed, and 100 µL of DMSO was added to each well to dissolve the formazan crystals. After 20 min incubation (5 % CO_2_, 37 ℃), the absorbance was measured at 540 nm using a microplate scanning spectrophotometer (ELISA reader, Organon Teknika, Boxtel, the Netherlands), and the cell viability was measured using the formula below.


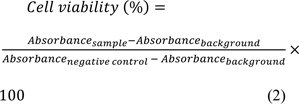


where negative control and background were the treated cells with only media, and only media, respectively. All experiments were performed in triplicate. 

#### Cell migration

To evaluate the effects of the standard ABZ and ABZ-loaded MCM-41 NPs on HepG2 cell migration, the cells were cultured in 12-well plates containing RPMI 1640 medium. At 90-95 % confluency, the scratches were introduced onto the monolayer cell surfaces by a 200 µL sterile pipette tip, and a cell-free area was developed. The cellular debris was removed by gentle washing using a culture medium, and the scratches were imaged (0 h). Next, the media were discarded, and the cells were treated with the standard ABZ and ABZ-loaded MCM-41 NPs at the drug concentrations of 25 µM. The plates were then incubated (5 % CO_2_, 37 ℃) for 24 h. The scratches were imaged after 24 h, and their area in each well was measured and analyzed. Finally, the efficacy of ABZ and ABZ-MCM-41 NPs to inhibit cell migration was calculated as a percentage using the following formula:


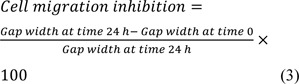


### Statistical analysis

All statistical analyses were performed using GraphPad Prism software version 8.00. ImageJ software (NIH, USA) was used to measure cell migration. Size, size distribution, drug loading efficiency, N_2_ adsorption, cell viability data, and cell migration date were expressed as the mean ± standard deviation (SD,* n *= 3). Statistical differences were analyzed by one-way analysis of variance (ANOVA) test. Statistical analysis was performed using nonlinear regression analysis, and comparisons were made for the half-maximal inhibitory concentration (IC_50_; the inhibitory concentration, which causes a decrease of 50 % in cell growth compared with untreated control) values utilizing Tukey's test.

See also the Supplementary data.

## Results and Discussion

### Characterization of nanoparticles

#### Dynamic light scattering

**Figure 2:**
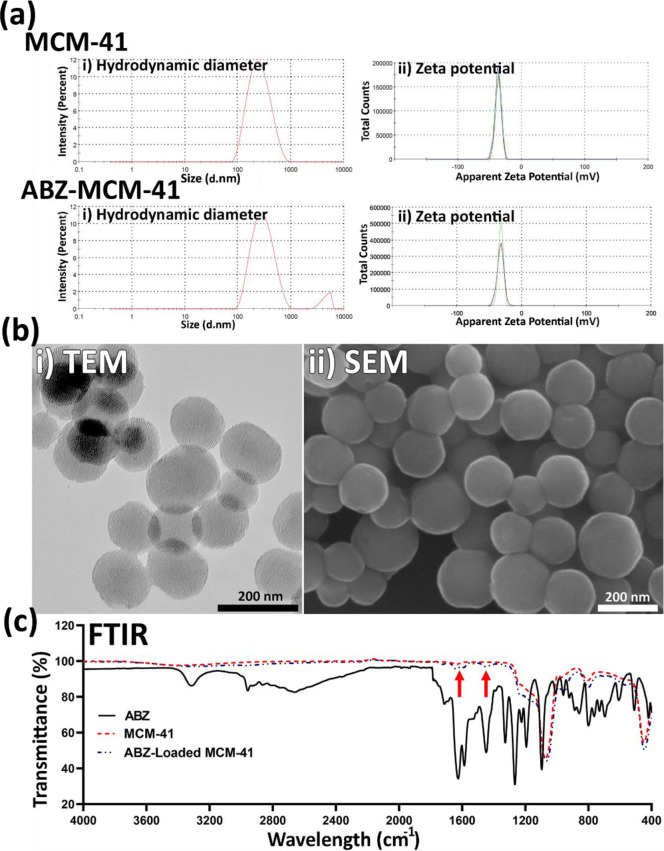
(a) i) Hydrodynamic diameter and ii) zeta potential of Mobil Composition of Matter No. 41 (MCM-41) and albendazole (ABZ)-loaded MCM-41 NPs, measured by the dynamic light scattering (DLS) method. As the results demonstrated, both NP formulations were formed at the nanoscale size (220 ± 11.5 and 293 ± 8.7 nm for MCM-41 and ABZ-loaded MCM-41 NPs, respectively). Also, as the Figure shows, both NPs had negative zeta potential equal to -36.3 ± 4.57 and -33.0 ± 4.93 mV, respectively. (b) i) Transmission electron microscopy (TEM) and ii) scanning electron microscopy (SEM) of ABZ-loaded MCM-41 NPs. As the TEM image demonstrates, mesoporous NPs with a hexagonal array of channels were formed. Also, the SEM image demonstrates that ABZ-loaded MCM-41 NPs were synthesized as homogeneous spheres without any discernible aggregation (×100,000 mag). (c) Fourier-transform infrared spectroscopy (FTIR) spectra of ABZ, MCM-41, and ABZ-loaded MCM-41 NPs. The peaks related to pure ABZ (e.g., 1623 cm^−1^, demonstrated by arrows) were observed in the spectrum of ABZ-loaded MCM-41 NPs, confirming that ABZ was loaded into the NPs. Also, as the ABZ-related peaks remained intact in ABZ-loaded MCM-41 NPs, it could be concluded that the drug was loaded into these NPs physically.

MCM-41 NPs were successfully synthesized using CTAB, as a precursor, and TEOS, as the silicate source. The synthesized NPs were first characterized in terms of size. The size of NPs is a determinant factor affecting the efficiency of loaded therapeutics in that smaller NPs are more internalized into cells, thereby increasing the intracellular concentration of the loaded therapeutics (Ghaferi et al., 2020[23]). NPs with a size below 300 nm are efficiently internalized into target cells and exhibit their pharmaceutical effects (Liu et al., 2014[44]). The resulting NPs, in the present study, were found with a size below 300 nm (220 ± 11.5 and 293 ± 8.7 nm, for MCM-41 NPs and ABZ-loaded MCM-41 NPs, respectively, Figure 2a(Fig. 2)) (Pandey et al., 2018[53]; Tzankova et al., 2019[67]). Also, size distribution is a critical factor that affects various properties of NPs, such as biological effects (Ho, 2014[29]), reproducibility (Syned, 2014[65]), and stability (Dhome et al., 2018[17]). Particles with varying sizes have various properties in terms of i) blood circulation, ii) cellular uptake, and iii) biodistribution (Ho, 2014[29]). Also, increasing the particle size and size distribution can result in the physical instability of NPs (Dhome et al., 2018[17]). The size distribution of MCM-41 and ABZ-loaded MCM-41 NPs were equal to 0.214 and 0.282, respectively, indicating that these NPs were homogenous and monodisperse (He et al., 2020[28]; Honary et al., 2013[30]). Also, the zeta potential of NPs is an important factor to determine the stability of NPs suspension as NPs with the same charge (positive or negative) in aqueous solutions with low ionic strength repulse each other, and this restrains their aggregation (Ghaferi et al., 2020[23]; Koohi Moftakhari Esfahani et al., 2021[39]). The zeta potential of MCM-41 and ABZ-loaded MCM-41 NPs were found to be -36.3 ± 4.57 and -33.0 ± 4.93 mV, respectively.

#### Transmission electron microscopy

TEM was used to evaluate the size and structural features of ABZ-loaded MCM-41 NPs. The results of TEM confirmed the results of DLS and demonstrated that the NPs were synthesized at the nanoscale size. As Figure 2b(Fig. 2) shows, the NPs were formed in uniform nanospheres. Also, the porous structure of these NPs can be clearly observed.

#### Scanning electron microscopy

SEM was used to evaluate the surface morphology and size distribution of ABZ-loaded MCM-41 NPs. The SEM image of the NPs demonstrated that these particles were synthesized as homogenous and spherical NPs, which had a smooth surface (Figure 2b(Fig. 2)). Also, it demonstrated that ABZ-loaded MCM-41 NPs were well dispersed without aggregation. 

#### Fourier Transformed Infrared Spectroscopy

Figure 2c(Fig. 2) demonstrates the FTIR of pure ABZ. The peak assigned to 3323 cm^−1^ was related to the stretching vibration mode of amide N-H. The absorption band observed at around 2960 cm^−1^ was related to the aliphatic hydrocarbon group (C-H). The ester C=O bond of the carbamate portion was observed at about 1713 cm^−1^. The peak related to the aromatic C=C bond was observed at around 1623 cm^−1^, which along with the amide N-H bond, constitute the benzimidazole portion of the drug. The peak observed at around 1523 cm^−1 ^indicated the stretching vibration mode of the C=N group (Adrover et al., 2020[2]). Overall, the FTIR spectrum indicated normal bands of pure ABZ (Adrover et al., 2020[2]). Also, the bands related to asymmetric and symmetric Si-O-Si stretches of MCM-41 were observed at 1110 and 825 cm^-1^, respectively. A peak at around 980 cm^-1^ was also related to the tension of the Si-OH bonds of the MCM-41 compound (Giraldo et al., 2019[25]). The existence of ABZ related peaks in ABZ-loaded NPs (e.g., 1623 cm^−1^, demonstrated by arrows in Figure 2c(Fig. 2)) confirmed that ABZ was loaded into the NPs. According to the results, ABZ preserved its chemical bonds in MCM-41 NPs, indicating the physical loading of ABZ into the NPs.

#### Thermogravimetric analysis and differential scanning calorimetry

**Figure 3:**
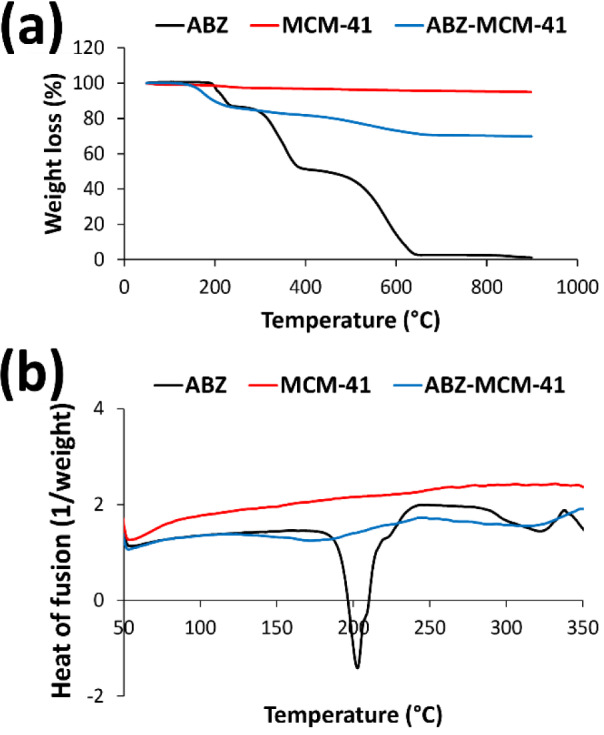
(a) Thermogravimetric analysis (TGA) thermograms of albendazole (ABZ), Mobil Composition of Matter No. 41 (MCM-41), and ABZ-loaded NPs and (b) differential scanning calorimetry (DSC) profiles of ABZ, MCM-41, and ABZ-MCM-41 NPs. Panel a shows, MCM-41 NPs experienced an initial weight loss at 250 °C, resulting in approximately 3 % weight loss. ABZ-loaded NPs were also decomposed at two temperatures, 150 and 450 °C, and this decomposition led to a decrease of 30 % in the mass of the formulation. In addition, ABZ was completely degraded when the temperature reached 900 °C. In panel b, pure ABZ showed a melting endothermic peak around 200 °C, while there were no obvious peaks in the thermogram of MCM-41 NPs. A melting endothermic peak but not sharp was also observed in the thermogram of ABZ-MCM-41 NPs at around 170 °C, which could be attributed to the crystalline anhydrous state of ABZ.

To determine the drug loading efficiency and thermal stability of ABZ, the TGA curves (mass vs. temperature) of ABZ, MCM-41, and ABZ-loaded MCM-41 NPs were measured. According to the results (Figure 3a(Fig. 3)), an initial weight loss occurred in MCM-41 NPs at around 250 ℃ due to the evaporation of absorbed water molecules, indicating the hydrophilic nature of this carrier, which is an advantage for loading of poorly water-soluble drugs (Khezri et al., 2014[36]). Also, ABZ-loaded MCM-41 NPs started to be degraded at 150 ℃ and continued up to 900 ℃. This resulted in a mass loss of 30 %, which was related to the degradation of ABZ. Based on the results, the amount of ABZ adsorbed onto the NPs was 30 %. ABZ was almost completely degraded at 900 ℃ (Figure 3a(Fig. 3)). 

DSC analysis can be used to investigate the existence or absence of a crystalline drug (e.g., ABZ) in the pores of mesoporous NPs (Alamdarnejad et al., 2013[3]; Jafari et al., 2016[33]). Also, this method can be used to study the glass transition temperature (T_g_) of the samples, where at the temperature above T_g_, various physical properties of a material, such as free molecular volume, heat capacity, thermal expansion coefficient, dielectric coefficient, and viscoelastic features, suddenly change (Bhandari and Howes, 1999[11]; Khezri et al., 2014[36]). Figure 3b(Fig. 3) illustrates the DSC profiles of the standard ABZ, MCM-41, and ABZ-loaded MCM-41 NPs. As MCM-41 NPs did not have any transitions in the temperature range of 50-350 ℃, only the thermal transition of ABZ was observed (Khezri et al., 2014[36]). Thus, a melting endothermic peak at around 200 ℃ was observed in the thermogram of ABZ, which is indicative of a crystalline anhydrous state of the drug. Also, the DSC profile of ABZ-MCM-41 demonstrated a melting endothermic peak around 170 ℃, which was related to the crystalline anhydrous state of ABZ, confirming drug loading into NPs.

#### Drug release from nanoparticles

Several daily doses of a drug are needed to attain and preserve the therapeutic concentration of the drug. This might lead to significant fluctuations in the plasma concentration of the drug (Fahr and Liu, 2007[20]), resulting in a decrease in the concentration beyond the minimum effective concentration, or an increase in the concentration above the minimum toxic concentration, and consequently, resulting in the lack of therapeutic effects or unfavorable toxic effects (Ghaferi et al., 2020[23]). These fluctuations in the plasma drug concentration can be reduced using sustained-release and controlled-release drug delivery systems, leading to improvements in the therapeutic outcome of the drug (Porta-i-Batalla et al., 2016[56]).

**Figure 4:**
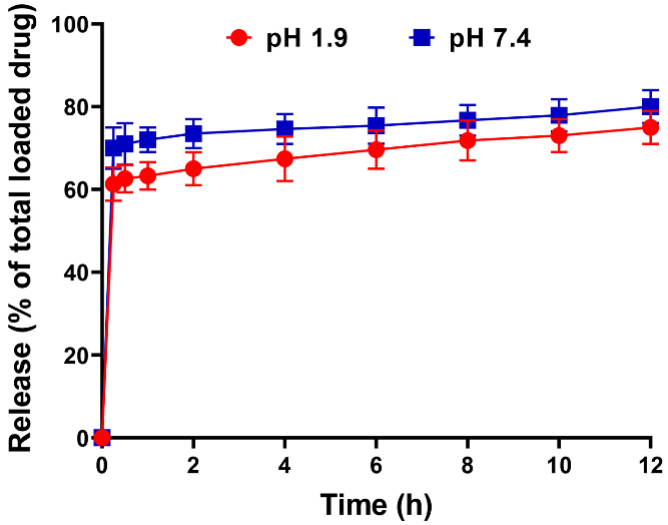
The cumulative percentage of albendazole (ABZ) release versus time from ABZ-loaded Mobil Composition of Matter No. 41 (MCM-41) NPs at pH 1.9 and 7.4. As the results showed, a burst drug release from the NPs occurred at the first 15 min of the study, leading to the release of 62 and 70 % of the loaded drug at pH 1.9 and 7.2, respectively. The release was then continued with a sustained and controlled release in the remaining time of the study. Statistical analyses were performed using one-way analysis of variance (ANOVA) and F-tests. The data are expressed as mean ± SD (n = 3).

In the present study, in order to simulate the pH changes in the gastrointestinal tract, the drug release was evaluated at pH 1.9 and 7.4, corresponding to the pH values of the human stomach (Kavousi et al., 2019[35]) and intestinal fluids (Chen et al., 2008[14]), respectively. The results demonstrated that the release of ABZ from MCM-41 NPs was initiated with a burst release, in which in the first 15 min of the study, 62 and 70 % of the loaded ABZ was released at pH 1.9 and 7.4, respectively (Figure 4(Fig. 4)). The burst drug release could stem from the release of the adsorbed drug onto the NPs or weakly bound between the drug and the NPs surface (Ghaferi et al., 2020[22]). In addition, by increasing the surface area of NPs, the amount of initial burst release increased (Alavi et al., 2019[10]). The pattern of the drug release continued with a gradually increasing trend at both pH values, in which 75 and 80 % of the loaded drug were released after 12 h, indicating a sustained and controlled drug release pattern.

Also, according to the results, the drug release was pH-dependent; however, the difference in the amount of the drug release between the two pHs was not statistically significant. These results were approximately similar to the results of Nguyen et al.'s study (2017[51]), where the difference in the amount of drug (prednisolone) release from MSNs at pH 1.9 and pH 7.4 was ~ 2 %. In the current study, the difference in the amount of drug release in two pHs could be related to various factors, such as MSNPs agglomeration at acidic pH and variation in the surface charge of the nanoformulation in different pH (Vazhayal et al., 2014[68]; Zeleňák et al., 2018[70]). At acidic pH (pH 1.9), the drug release from the pores of the agglomerated particles is inhibited, resulting in lower drug release (Zeleňák et al., 2018[70]). Also, to determine the kinetics of the drug release, the profiles of drug release were analyzed using different kinetic models, including zero and first order, Higuchi, and Korsmeyer Peppas models, and the correlation coefficient values were determined for the linear curves. Based on the results, the higher R^2^ values (0.3766 and 0.4156 at pH 1.9 and 7.4, respectively) were obtained in the Higuchi model compared to other drug release models; thus, the drug release from the nanoformulation at both pH followed the Higuchi kinetic model.

#### Brunauer-Emmett-Teller surface area analysis

**Figure 5:**
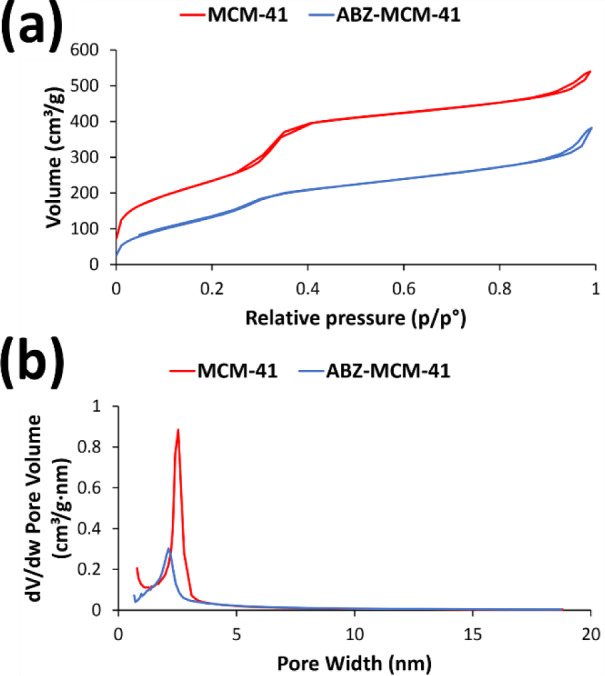
N2 adsorption/desorption isotherms of Mobil Composition of Matter No. 41 (MCM-41) and albendazole (ABZ)-MCM-41 NPs for measuring their (a) surface volume and (b) pore size. As the results showed, a sharp increase in the N2 uptake around p/p* 0.3 occurred, which could be attributed to capillary condensation in cylindrical pores of MCM-41 and ABZ-loaded MCM-41 NPs. Also, the surface volume and pore size of the carrier decrease after drug loading. However, this decrease does not affect the mesoporous structure of this carrier.

The N_2_ adsorption/desorption isotherms of the calcined MCM-41 and ABZ-loaded MCM-41 NPs are demonstrated in Figure 5(Fig. 5). MCM-41 and ABZ-loaded MCM-41 NPs demonstrated the behavior of the mesoporous material and type IV isotherm based on the IUPAC isotherm classification system. These isotherms can be divided into three steps, including i) a linear increase in N_2_ adsorption, occurring at relatively low pressure owing to the monolayer adsorption of N_2_ on the wall of MCM-41 and ABZ-loaded MCM-41 NPs; ii) capillary condensation of N_2_ inside the mesopores, which is indicative of narrow pore size distribution; and iii) saturation step, presented as a long plateau at higher pressures owing to the low N_2_ adsorption onto the external surface of the calcined MCM-41 and ABZ-loaded MCM-41 NPs (Khezri et al., 2014[36]). Based on these results, the surface volume for MCM-41 and ABZ-loaded MCM-41 NPs was found to be 540 and 380 m^2^/g, respectively, while the pore size for these formulations was found to be 2.5 and 2.1 nm, respectively. In addition, the pore volume of MCM-41 and ABZ-loaded MCM-41 NPs was determined to be approximately 0.75 and 0.46 cm^3^/g, respectively.

### Biological effects of the nanoparticles

#### Cytotoxicity studies

**Figure 6:**
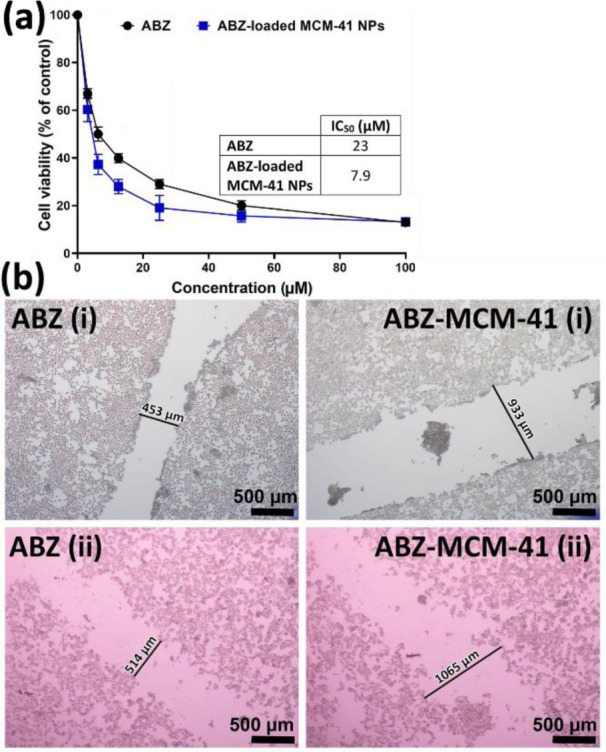
(a) Cell viability effects of albendazole (ABZ) and ABZ-loaded Mobil Composition of Matter No. 41 (MCM-41) NPs on HepG2 liver cancer cells after 24 h. As the Figure demonstrates, the loading of ABZ into MCM-41 NPs caused a significant reduction in HepG2 cell viability compared to that of the standard drug (P<0.05). The data were expressed as mean ± SD (n = 3). (b) Effects of ABZ and ABZ-loaded MCM-41 NPs on the HepG2 cells migration i) before (0 h) and ii) after (24 h) cell treatment (×40 mag). Both ABZ and ABZ-loaded MCM-41 NPs restrained the cell migration by approximately 12 %, confirming the cytotoxicity effects of ABZ and ABZ-MCM-41 NPs.

The cytotoxicity effects of ABZ, MCM-41, and ABZ-loaded MCM-41 NPs were investigated on HepG2 cells using MTT assay to determine if ABZ loading into NPs caused enhanced cytotoxicity effects or not. For this purpose, ABZ at the concentrations of 0, 3.1, 6.3, 12.5, 25, 50, and 100 µM were used as these concentrations encompass the reported serum concentration of ABZ (4.3 µM), when administered at the standard doses (10 mg/kg/day). Also, a significantly higher drug concentration (100 µM) was used to make the toxicity more pronounced. The results demonstrated that MCM-41 NPs, at the concentration of 25 µg/mL, had no toxicity effects on the cells. It was found that ABZ loading into NPs caused a significant increase in the cytotoxicity effects of the drug in a concentration-dependent manner (Figure 6a(Fig. 6)), in which the IC_50_ values for ABZ and ABZ-MCM-41 NPs were estimated 23 and 7.9 µM, respectively.

#### Cell migration

Migration is a distinctive feature of cellular behavior that contributes to embryogenesis, tissue remodeling, wound healing, and pathologies, such as cancer metastasis and invasion (Glenn et al., 2016[26]; Grada et al., 2017[27]). The cell migration was determined using HepG2 cells at different time intervals to recognize the distance at which cancer cell invasion happened. A scratch was generated and treated with ABZ and ABZ-loaded MCM-41 NPs at the drug concentration of 25 µM. The cancer cell migration was monitored by taking pictures at times 0 and 24 h (Figure 6b(Fig. 6)). As the results demonstrated, both ABZ (gap width of 453 and 514 µm at times 0 and 24 h, respectively) and ABZ-loaded MCM-41 NPs (gap width of 933 and 1065 µm at times 0 and 24 h, respectively) inhibited cell migration by approximately 12 %. This indicated that, after loading ABZ with MCM-41 NPs, its potency to inhibit cell migration was unchanged.

## Conclusion

Poorly water-soluble drugs have low absorption, and consequently, have low bioavailability (Chaudhari and Handge, 2020[13]), which causes a considerable decrease in the therapeutic response and an increase in the overall dose (Abu-Huwaij, 2018[1]). ABZ has poor water solubility, and as a result, has low bioavailability (Savio et al., 1998[63]). MSNPs are able to increase the solubility rate of poorly water-soluble drugs (Adrover et al., 2020[2]; Ghaferi et al., 2021[24]). The present study aimed to increase the efficacy of ABZ against liver cancer cells through loading into MCM-41 NPs. The MCM-41 and ABZ-loaded MCM-41 NPs were successfully synthesized using CTAB as a precursor. The characterization results demonstrated that nanoscale size particles were constructed. Both NPs were monodisperse with high thermal stability. MCM-41 NPs demonstrated high potency for the loading of ABZ (with the drug loading efficiency of 30 %), which is a poorly water-soluble drug. This can cause a significant increase in ABZ solubility and, as a result, drug bioavailability. Also, ABZ-loaded MCM-41 NPs can significantly increase the cytotoxicity effects of ABZ (by 2.6-fold) against liver cancer HepG2 cells. Also, the cell migration results demonstrated that both ABZ and ABZ-MCM-41 NPs could inhibit cancer cell migration by approximately 12 %. Overall, the results of the present study suggest evaluating the efficacy of ABZ-loaded MCM-41 NPs *in vivo* environment.

## Notes

Hasan Ebrahimi Shahmabadi and Seyed Ebrahim Alavi (Department of Microbiology, School of Medicine, Rafsanjan University of Medical Sciences, Rafsanjan, Iran; Postal code: 7717933777, Tel: +983431315043, E-mail: s.ebrahimalavi@gmail.com) contributed equally as corresponding authors.

## Declaration

### Authors' contribution

Conceptualization, M.G., S.E.A., A.A. and H.E.S; methodology, W.Z.; software, A.A.; validation, S.E.A. and M.G.; formal analysis, M.G.; investigation, W.Z. and A.A.; writing-original draft preparation, H.E.S. and S.E.A.; writing-review and editing, S.E.A.; visualization, S.E.A.; supervision, S.E.A. and H.E.S.; project administration. All authors have read and agreed to the published version of the manuscript.

### Conflict of interest

The authors declare that they have no conflict of interest.

### Informed consent

This article does not contain any studies with human participants.

## Supplementary Material

Supplementary data
